# Potential Opportunities for Pharmacogenetic-Based Therapeutic Exploitation of Xanthine Dehydrogenase in Cardiovascular Disease

**DOI:** 10.3390/antiox13121439

**Published:** 2024-11-22

**Authors:** Gianmichele Massimo, Nicki Dyson, Fisayo Olotu, Rayomand S. Khambata, Amrita Ahluwalia

**Affiliations:** Barts & The London Faculty of Medicine & Dentistry, Queen Mary University of London, Charterhouse Square, London EC1M 6BQ, UK; g.massimo@qmul.ac.uk (G.M.); n.dyson@qmul.ac.uk (N.D.); f.olotu@qmul.ac.uk (F.O.); r.s.khambata@qmul.ac.uk (R.S.K.)

**Keywords:** xanthine oxidoreductase, cardiovascular diseases, glycosaminoglycans, NO, nitrite, nitrite reduction

## Abstract

The majority of naturally occurring mutations of the human gene *XDH*, are associated with reduced or completely absent xanthine oxidoreductase (XOR) activity, leading to a disease known as classical xanthinuria, which is due to the accumulation and excretion of xanthine in urine. Three types of classical xanthinuria have been identified: type I, characterised by XOR deficiency, type II, caused by XOR and aldehyde oxidase (AO) deficiency, and type III due to XOR, AO, and sulphite oxidase (SO) deficiency. Type I and II are considered rare autosomal recessive disorders, a condition where two copies of the mutated gene must be present to develop the disease or trait. In most cases, xanthinuria type I and II result to be asymptomatic, and only occasionally lead to renal failure due to urolithiasis caused by xanthine deposition. However, in the last 10–15 years, new observations have been made about the link between naturally occurring mutations and pathological phenotypes particularly pertinent to cardiovascular diseases (CVD). These links have been attributed to a genetically driven increase of XOR expression and activity that is responsible for what is thought to be damaging uric acid (UA) and reactive oxygen species (ROS) accumulation, nitric oxide (·NO) depletion and endothelial dysfunction. In this review, we discuss the importance of genetics for interindividual variability of XOR expression and activity while focusing mainly on those variants thought to be relevant for CVD. In addition, we discuss the potential exploitation of the genetically driven increase of XOR activity to deliver ***more*** beneficial bioavailable ·NO. Finally, we examine the effect that non-synonymous mutations have on the tertiary structure of the protein and consequently on its capacity to interact with glycosaminoglycans (GAGs) localised on the outer surface of endothelial cells.

## 1. Introduction

**Figure 1:**
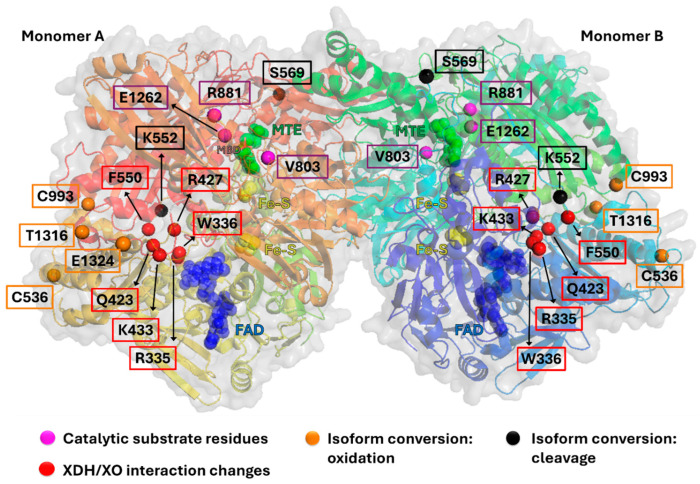
Position of key functional residues in XOR. Purple Residues: Required for functional enzymatic molybdenum dependent activity. Red Residues: interaction between these residues is lost in the XDH to XO isoform conversion. Orange Residues: sites of cystine oxidation, which induces XDH to XO isoform conversion. Black Residues: sites of proteolytic cleavage which induces XDH to XO isoform conversion. Figure derived from human XOR crystal structure (PDB 2E1Q). Also shown are (Green) substrate MTE (phosphonic acidmono-(2-amino-5,6-dimercapto-4-oxo-3,7,8a,9,10,10a-hexahydro-4h-8-oxa-1,3,9,10-tetraaza-anthracen-7-ylmethyl)ester) in the molybdenum active pocket, (Yellow) Fe-S clusters, (Blue) FAD in the FAD domain, and (MBD) molybdenum.

Xanthine oxidoreductase (XOR) is encoded by the human gene *Xanthine dehydrogenase* (*XDH*). However, mammalian XOR exists in two biochemical isoforms, XDH and xanthine oxidase (XO), whereas non-mammalian organisms only have the dehydrogenase form. The enzyme is ubiquitously expressed (mRNA identified in most tissues/cells); however, activity levels are low in most tissues with high expression levels being evident in few cell types/tissues (reviewed in [1]). The highest expression and activity levels are found in the liver (hepatocyte), which is recognised to be the main expressing site, with the small intestine and mammary glands (during lactation) also being key sites of expression/activity [2,3,4,5,6]. Numerous findings have shown that under diverse pathological settings, not least including ischaemia-reperfusion(I/R) [1,7], inflammation [8,9,10] and hypertension [11,12] the hepatic expression and activity of the enzyme are significantly increased. This is followed by substantial release of XDH into the general circulation, where it is converted to the oxidative isoform, XO, either reversibly via oxidation of cysteine residues (Cys536, Cys993, Cys1316, Cys1324) or irreversibly via proteolytic cleavage of lysine (Lys552, Lys569), respectively [13,14,15] (Figure 1). It is also apparent that non-synonymous mutations of the human gene *XDH* seem to be responsible for pre- and post-translational modifications, influencing not only expression but also XOR levels and the proportion of XDH versus XO [16].

**Figure 2:**
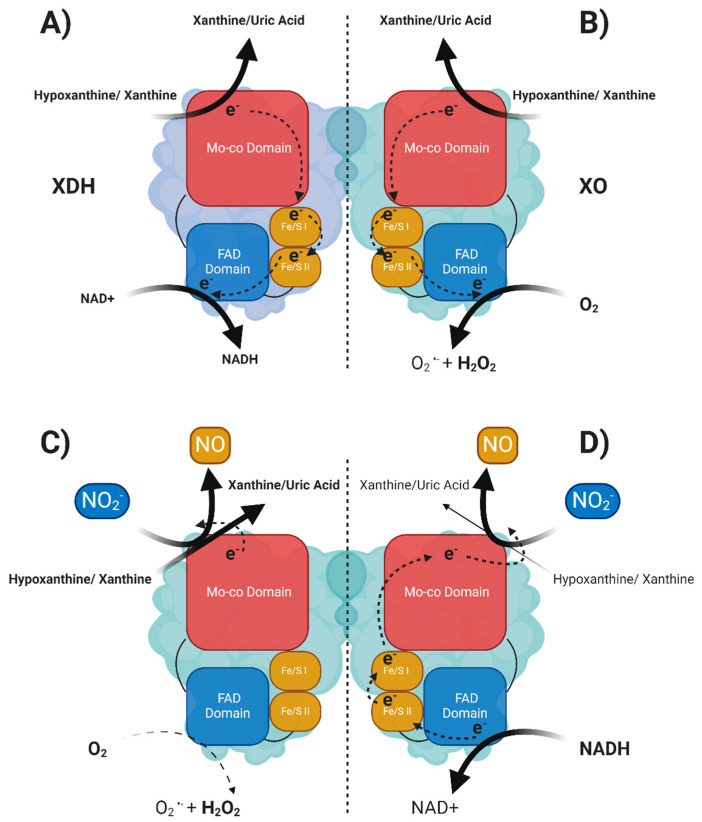
Schematic of XOR homodimer structure and the catalysed reactions and electron flux within an XOR monomer. (**A**) XOR monomer in the *XDH* state oxidises purines at the Mo-co domain. The electrons at the reduced Mo-co domain are shuttled to the FAD domain via the Fe/S clusters restoring the Mo-co domain oxidation state (Mo^+4^ to Mo^+6^). The *XDH* isoform has an increased affinity to utilise NAD+ as a terminal electron acceptor. (**B**) XO has an increased affinity for O_2_ as a terminal electron acceptor and produces O_2_^•−^ and H_2_O_2_. (**C**) Electrons donated from purines oxidation are utilised by the Mo-co domain to reduce NO_2_^−^ which prevents the formation of O_2_^•−^ and H_2_O_2_ at the FAD domain (this reaction is favoured in low O_2_ concentrations). (**D**) NADH acts as a reducing substrate and donates electrons at the FAD domain which, via retrograde electron flux, reduces the Mo-co domain. The Mo-co domain can reduce NO_2_^−^ to NO, which resolves its oxidation state. This NADH reduced Mo-co domain favours NO_2_^−^ and reduces UA production (this reaction favours high NADH concentrations and acidic pH).

Certainly, liver XOR has a broad oxidising activity and a low specificity for endogenous and exogenous substrates, drugs included: activity that underlies a key detoxification process within the liver. But perhaps the most well-accepted role of the enzyme is in its function to catalyse the last two steps of purine metabolism, which consists of two consecutive hydroxylation reactions of hypoxanthine to xanthine and xanthine to uric acid (UA) (Figure 2). The hydroxylation mechanisms have been extensively reviewed by Nishino and colleagues and we refer the readers to these excellent articles [17,18,19]. It is now well accepted that few amino acids of the active site, glutamic acids (Glu) 1262 and 803, as well as arginine (Arg) 881, play a key role in catalysing purine hydroxylation. Glu1262 is responsible for initiating the enzymatic reaction by deprotonating the Mo-OH group, which in turn will direct a nucleophilic attack of C-2 of the substrate. On the contrary, Arg803 and Arg881 are involved in purine binding and stabilisation. These findings were confirmed by site specific mutagenesis experiments, where replacement of Glu1262 generated an enzyme completely devoid of any hydroxylation capacity [19]. In addition, substitution of amino acid residues of the active site of XOR, Arg803 or Arg881, with valine (Val) and methionine (Met) generated enzymes with almost no detectable activity toward hypoxanthine or xanthine, respectively. However, both mutants displayed an Aldehyde Oxidase (AO)-like activity [19].

Whilst both XDH and XO isoforms have the capacity to oxidise purines at the molybdenum-pterin (Mo-Pt) binding site, where two electrons are generated for each molecule of substrate and transferred to the enzyme (Mo^+VI^ to Mo^+IV^), differences appear at the flavin adenine dinucleotide (FAD) domain where two different electron acceptors, nicotinamide adenine dinucleotide (NAD^+^) or dioxygen (O_2_), may be used according to the oxidative state of the enzyme. It is well accepted that regardless of the mechanism responsible for the conversion, reversible or irreversible, the transition from XDH to XO is accompanied by a substantial intramolecular reorganisation of the enzyme due to a loss of interaction between key amino acids, Phe550, Arg335, Trp336, and Arg427, which form a critical relay system. A tight interaction between these amino acid residues stabilises the enzyme in its dehydrogenase state with the active loop (Gln423–Lys433), allowing NAD^+^ to approach the FAD domain where it is reduced to NADH (although XDH can also use O_2_ in the setting of NAD^+^ deficiency). On the contrary, the oxidative or the proteolytic conversion of XDH to XO is accompanied by a loss of interaction between Trp336 and Phe550 which is sufficient to induce a conformational change of the active loop, preventing access for NAD^+^ to the FAD domain and allowing O_2_ to enter the solvent channel [20,21,22,23]. O_2_ can then be reduced by the enzyme to O_2_^−^ or to H_2_O_2_ via one or two electron reduction reactions, respectively.

It is worth noting that pathological settings such as I/R, characterised by low oxygen tension, are accompanied by alterations of the mitochondrial respiratory chain with consequent accumulation of metabolic intermediates in the form of reducing equivalents and protons. In particular, hypoxia leads to raised levels of NADH due to reduced mitochondrial NADH oxidation, and in such circumstances XOR, mainly in the XDH state, exhibits a highly effective NADH oxidase activity at the FAD site, which is completely independent from the Mo-Pt domain, and is blocked by the FAD site inhibitor diphenyleneiodonium (DPI) [24]. Studies inducing chemical modification of the amino acid residue Tyr393 with fluorosulfonylbenzoyl adenosine (FSBA) led to the generation of an enzyme void of NAD^+^ activity, suggesting that this amino acid is critical for binding NADH [25]. Electrons produced by NADH oxidation may be either transferred via the FAD domain (reduced to FADH_2_) to O_2_, during reperfusion, generating O_2_^•−^ and H_2_O_2,_ or intramolecularly via the two iron-sulphur cluster domains (Fe-S I,II) to the Mo-Pt domain (which passes from Mo^+VI^ to Mo^+IV^) where they can be used for the most recently described “unconventional” activity of the enzyme of reducing NO_2_^−^ to beneficial NO [24,26,27]. It is this XOR-dependent ·NO formation that has been implicated in the recently dubbed non-canonical pathway of ·NO synthesis [28]. This NO_2_^−^ -reductase activity of XOR, irrespective of the electron donors available (hypoxanthine/xanthine or NADH), catalyses a one-electron reduction of NO_2_^−^ to ·NO and below we provide an update on this newest aspect of XOR activity.

## 2. Biochemical Mechanisms of XOR-Dependent NO_2_^−^ Reduction

Despite the only very recent appreciation of the NO_2_^−^ reductase aspect of XOR activity, the nitrate-reducing activity of the enzyme was first demonstrated in 1924 by M. Dixon and S. Thurlow. They used milk-derived or purified XO, demonstrating reduction of nitrate (NO_3_^−^) to NO_2_^−^ [29]. It was, however, not until 1998 that Millar [30] and Zhang [27] simultaneously published their observations demonstrating that both purified XOR and tissue homogenates, containing XOR, also reduce NO_2_^−^ to ·NO. Millar et al. did not detect any chemiluminescent signal in the presence of O_2_, suggesting that the reaction needed NADH as an electron donor and that ambient O_2_ concentrations suppressed activity [30]. On the contrary, Zhang et al. demonstrated that this reaction not only takes place in a hypoxic environment (K_m_ = 2.4) but also in the presence of ambient O_2_ (K_m_ = 2.3), exhibiting similar sigmoidal curves [27]. Moreover, using pharmacological inhibitors of XOR activity i.e., allopurinol and DPI, targeting the Mo-Pt and FAD domains, respectively, the authors also demonstrated that this reaction takes place at the Mo-Pt domain. A further extensive analysis of the NO_2_^−^ reductase activity of XOR has been completed by Jay Zweier and his group [31,32,33]. Zweier’s observations were in line with Millar et al. [30]. However, in addition it was shown that higher concentrations of purines (>20 µM) inhibit NO_2_^−^ reduction, explaining this phenomenon by suggesting an impact of steric hindrance at the Mo-Pt site.

The molecular mechanism of NO_2_^−^ reduction operated by molybdoenzymes has been extensively investigated and discussed by Luisa Maia et al. [34,35]. The authors demonstrated that both XDH and XO have a similar rate of ·NO production, measured using a selective NO-electrode [34]. Purine oxidation and NO_2_^−^ reduction were suggested to be two consecutive reactions which follow a precise time order. Considering that both reactions take place at the Mo-Pt site, the authors theorised that NO_2_^−^ interacts with a fully reduced Mo-Pt^(+IV)^ domain only once urate has been released. Specifically, NO_2_^−^ would interact through one of its two O atoms to the Mo-Pt^(+IV)^ domain, and the same O atom would then undergo a protonation step resulting in a homolytic O-N bond cleavage and formation of one ·NO molecule. ·NO would then leave the semi-reduced Mo-Pt^(+V)^ domain, which would then be ready to catalyse the reduction of a new molecule of NO_2_^−^, leading to the formation of a second molecule of NO and fully restoring its oxidation state (Mo-Pt^(+VI)^) [35].

Glu1262, which is one of the three residues of the active pocket initiating the purine oxidation reaction, has been suggested to play a critical role in the reduction of NO_2_^−^. Although not experimentally demonstrated, it seems that Glu1262 holds favourable characteristics, mainly derived from its position within the active pocket, which make it a plausible proton (H^+^) donor necessary for freeing ·NO from the Mo-Pt domain [35]. The suggested protonation step required for breaking the O-N bond seems to be supported by observations obtained from the kinetic evaluation of the NO_2_^−^ reductase activity, according to which this reaction is significantly increased in an acidic environment [31,34,36,37]. If we consider that acidosis is a direct consequence of hypoxia [38], a condition where both XOR expression and activity are significantly upregulated [30,31,37,39,40,41], the potential physiological impact for this pathway for NO delivery within the cardiovascular system is considerable. This is particularly so since eNOS-dependent ·NO production is impaired under hypoxic conditions. The crosstalk between the canonical and non-canonical pathways has been demonstrated in several pre-clinical studies. Peleli et al. demonstrated that in eNOS knockout mice, a significant increase in XOR expression and activity, measured as ROS and UA production, was accompanied by an increased NO_2_^−^-reductase capacity compared to wild type mice [42]. We have also demonstrated that XOR-dependent (allopurinol-inhibitable) NO_2_^−^ reductase activity and consequently NO_2_^−^-induced BP lowering is increased in spontaneous hypertensive rats (SHR); importantly, the SHR is a model in which eNOS is dysfunctional [11].

There is now considerable data indicating the positive effects of XOR-driven NO_2_^−^ bioactivation in restoring ·NO levels in diverse pathological settings particularly characterised by endothelial dysfunction and, accordingly, reduced eNOS activity. This includes models simulating stroke [43], peripheral atherosclerosis [44], myocardial I/R [36], heart failure (HF) [12], and hypertension [11]. However, despite this, the role of XOR, good or bad, in CVD is controversial. This is due, not least, to its described activity of generating ROS and UA, products that have been clearly linked with disease progression and defined as key risk factors for CVD; this aspect of activity has been extensively reviewed elsewhere [45]. In addition, known risk factors for CVD such as ischaemia, hypertension, inflammation, cigarette smoke, atherosclerosis, obesity, and diabetes, are known to positively modulate XOR expression and activity.

Besides these modifiable risk factors in CVD, non-modifiable risk factors such as race and ethnicity also exist and seem to play a critical role in modulating XDH at pre- and post-translational levels. These latter influencers intimate that genetics are an important factor to consider when assessing individual XOR activity and its functional impact. Although numerous naturally occurring mutations in the h*XDH* gene have been identified, most of them have been associated with the reduction or complete abrogation of XOR activity, with a focus on ROS generation and purine degradation. Recently, our own lab has begun to explore what these mutations might mean for enzyme activity in the context of its NO_2_^−^-reductase activities [16]. This review focuses upon describing the pathophysiological implication of XDH mutations so far isolated, and particularly on those accompanied by raised enzymatic activity or associated with CVD and discusses what this might mean for its NO_2_^−^-reductase activity. We speculate that a better understanding of whether specific variants lead to altered pro-oxidative and uricaemic genotypes might lend to better stratification of treatments focussed upon elevating beneficial ·NO levels, leading to health benefits.

## 3. XOR in CVD

It is now well-accepted that raised sUA levels are associated with an elevated risk of certain CVD, and this characteristic is taken to indicate an increased XOR activity but also an increase in the provision of substrate. In vivo purine levels are derived both from endogenous synthesis and dietary sources, with primary contributors including meat and legumes [46,47,48]. Additionally, fructose, a common sweetener found in various beverages, serves as an important indirect dietary source that significantly stimulates in vivo UA synthesis via purine oxidation pathway [49]. Physiological circulating levels of serum UA (sUA) range between 1.5 and 6 mg/dL (0.09–0.36 mmol/L) in women and 2.5–7 mg/dL (0.15–0.42 mmol/L) in men [50,51,52]. These levels are maintained through a balance of production, degradation, reabsorption, excretion, and secretion, primarily in the renal proximal tubule and gastrointestinal tract [53]. Thus, lifestyle habits and particularly diet have a major influence over the apparent in vivo XOR activity levels. Consumption of purine-rich foods will inevitably raise sUA levels. When sUA levels exceed 6–7 mg/dL, this condition is classified as hyperuricemia, whereas levels below 2.0 mg/dL indicate hypouricemia. What is known is that, in addition to diet, genetic factors play a critical role in deviations such as in the well-documented inherited disorders such as renal hypouricemia and xanthinuria. However, understanding whether XOR mutations impact cardiovascular health is less well understood. In addition, whether these mutations are associated with changes in the levels of one or all of the products of XOR activity, i.e., UA, ROS production, and ·NO has largely not been explored. We speculate that work in this area is of potential interest in order to understand whether interventions specifically targeting the levels of these products (either with small molecules or through regulating substrate levels), might offer novel opportunities for therapeutics.

Perhaps the CVD that has been most strongly linked with altered XOR activity is hypertension. Hypertension is one of the most important risk factors for mortality worldwide [54]. Over the last two decades numerous pre-clinical and clinical studies have demonstrated that high levels of circulating UA correlate with BP. For instance, Cicero et al. found that sUA levels were significantly higher in untreated or uncontrolled hypertensive patients compared to normotensive or controlled hypertensive individuals [50]. In another study, Cicero et al. showed a positive correlation between sUA quartiles and the prevalence of hypertension, finding that sUA was independently associated with carotid intima-media thickness, a proxy for sub-clinical atherosclerosis [55]. In line with these observations, findings obtained from the Italian Project PIUMA (Progetto Ipertensione Umbria Monitoraggio Ambulatoriale) revealed that, following adjustment for well-known risk factors for CVD (such as age, sex, diabetes, lipid levels, serum creatine, left ventricular hypertrophy (LVH), and BP) sUA (at least for the highest two quartiles) was strongly associated with increased risk of CV events compared with those with sUA in the lower quartiles [56]. Additionally, the URRAH study (Uric Acid Right for Heart Health), a multicentre retrospective observational study, seeking to determine whether a threshold of sUA in hypertensive patients predicted risk of all-cause and CV mortality has suggested that the cut-off for sUA levels is 4.7 mg/dL and 5.6 mg/dL, respectively. Interestingly, these levels are both lower than the levels considered physiological (5.6–6.3 mg/dL) [57], suggesting that perhaps a more aggressive strategy to lower sUA would be of benefit. While clinical studies link UA to hypertension and other cardiovascular risks, the biochemical implications of specific *XDH* mutations on these mechanisms are underexplored. Identifying how such mutations influence XOR enzymatic activities could reveal pathways beyond UA production that contribute directly to cardiovascular outcome.

Despite all of the evidence linking sUA with hypertension, mendelian randomisation studies have failed to demonstrate a cause–effect relationship [58,59]. However, a key issue regarding the previous work is that the gene SNPs considered were predominantly limited to those linked with renal excretion rather than UA synthesis, potentially underestimating the role of UA in disease [60,61,62,63,64]. Moreover, the evidence interrogating the link between variants of the XDH gene and CVD was limited. Below we discuss the evidence to date and potential functional ramifications.

## 4. Genetic Disorders Related to Uric Acid Metabolism

Genetic variations in UA metabolism, particularly those affecting XOR, potentially impact upon the influence of XOR on cardiovascular health. Inherited hypouricemia arises from two main autosomal recessive disorders: renal hypouricemia and xanthinuria. Renal hypouricemia is further divided into two types. Type I, a rare hereditary disease, is caused by a loss-of-function mutation in the SLC22A12 gene, which encodes the human urate transporter 1 (URAT1), leading to reduced urate reabsorption in the renal proximal convoluted tubule. The second is type II caused by genetic mutations of the SLC2A9 gene encoding for the glucose-transporter 9 (Glut9), which is an efflux transporter responsible for urate secretion from the intracellular space of tubular cells to the interstitial space. Patients affected by inherited renal hypouricemia are mainly asymptomatic, however, severe complications such as exercise-induced acute kidney injury (EIAKI) and urolithiasis may appear. Inherited renal hypouricemia has been extensively reviewed by Hirotaka Matsuo and colleagues and we refer the readers to relevant articles [65]. Interestingly, to date, a link of these variants with CVD has been elusive.

Xanthinuria, on the other hand, results from defective XOR enzyme function. This genetic disorder is characterised by hypouricemia, elevated circulating xanthine levels, and xanthine excretion in the urine [66]. Xanthinuria is classified into three types: type I, caused by XOR deficiency; type II, due to mutations affecting both XOR and aldehyde oxidase (AO) [67]; and type III, which involves mutations in molybdenum cofactor sulfurase (MOCOS), impacting XOR, AO, and sulphite oxidase (SO) activity (all molybdenum containing enzymes). These classifications allow for a nuanced view of how specific enzymatic deficiencies within XOR pathways might relate to CVD risks. Classical xanthinuria, which includes types I and II, is a rare disorder with an estimated incidence of 1/69,000, while renal hypouricemia is more prevalent, as indicated by one study that identified eight hypouricemic individuals in a cohort of 1875 healthy Japanese subjects [68,69]. Such variations in incidence rates raise interesting questions about genetic predisposition and the potential variable impact of UA metabolism on cardiovascular health.

Global *Xdh* knockout mice (*Xdh*^−/−^) were developed to model classical xanthinuria type I, but these studies revealed significant limitations due to the lethality of the mouse model [70]. *Xdh^−/−^* mice appear normal at birth with no overt physical differences versus *Xdh^+/+^* littermates. However, *Xdh^−/−^* mice exhibited stunted growth and died within 2–4 weeks [70]. Postmortem examination of the *Xdh^−/−^* mice revealed that the kidneys were smaller, paler, and malformed. The renal insufficiency observed in these mice was accompanied by increased levels of blood urea nitrogen and extremely low sUA levels. In another study, the same group identified accumulation of triglycerides in the renal tubules of *Xdh^−/−^* mice, accompanied by deposition of xanthine crystals, inflammation, hypoxia, and raised levels of ROS, resulting in interstitial fibrosis [71]. Chen et al. confirmed these findings, also showing that the genetic ablation of XOR caused an end-stage kidney failure accompanied by interstitial fibrosis, and high and low circulating levels of creatinine and UA, respectively [72]. In addition, the authors reported that *Xdh^−/−^* mice were characterised by raised circulating levels of primary purine metabolites, hypoxanthine and xanthine, which were converted via the purine nucleoside phosphorylase 1 (PNP1) enzyme into secondary metabolites such as xanthosine, inosine, and adenosine. Considering that PNP1 is also responsible for the inter-conversion of deoxyuridine to uracil and N-ribosyl-nicotinamide to nicotinamide, the abundant presence of primary purine metabolites interferes with the physiological metabolism of these molecules, resulting in altered levels of pyrimidines and nicotinamide. The same pattern was also observed in mice treated with allopurinol [72]. The impact of *Xdh* deletion upon cardiovascular function, of course, with the 4 week lethality was difficult to determine; however, in heterozygous mice (*Xdh^+/−^*) where renal function was unaffected and mice survived through adulthood, some indication of raised BP was shown, associated with impaired vasodilator vessel reactivity [73].

In contrast to the severe phenotype observed in *Xdh^−/−^* mice, xanthinuria type I in humans presents with much milder symptoms, such as sporadic urolithiasis, acute renal failure, and myositis. Interestingly, while hypouricemia is generally benign, a reduction in sUA levels has been associated with an increased risk of CV events, revealing a J-shape curve for UA and CV risk [56]. Despite these observations, the relationship between hypouricemia and endothelial function is not fully understood. For instance, a study published in 2015 by Sugihara et al. reported reduced flow-mediated dilation (FMD) in hypouricemic patients, indicating potential endothelial dysfunction [74]. However, a case report found that a patient with xanthinuria type I demonstrated a normal FMD response, suggesting preserved endothelial function [75]. These contradictory findings highlight the need for further investigation into the link between hypouricemia and vascular health.

Since 1954, when the first case of inherited xanthinuria was reported [66], numerous mutations or SNPs associated with absent or reduced XOR activity have been identified (Table 1). Whilst the majority of these mutations are non-synonymous, e.g., the nucleotide substitution leads to the formation of a different codon encoding for a new amino acid, a few (p.Cys48Leufs*12, p.Pro214Glnfs*4, Leu305*1, p.Asp553Alafs*17, p.Ala556Serfs*15, and p.Thr856Lysfs*73) result from an insertion/deletion mutation causing a downstream frame shift and a potential introduction of a stop codon leading to an altered or truncated protein. Most of the identified mutations are located within the largest C-terminal domain, which includes the Mo-Pt binding site spanning from residues 591 to 1317 (Figure 1). Amino acid substitution within this region may lead to a spatial rearrangement of the Mo-Pt domain with a consequent alteration of its capacity to interact with the reducing agents, hypoxanthine and xanthine, resulting in a complete or partial loss of activity. Overall, while xanthinuria type I may lead to hypouricemia, the effects on cardiovascular health remain unclear, and additional studies are warranted to better understand the relationship between XOR activity, purine metabolism, and cardiovascular outcomes.

## 5. Naturally Occurring Human *XDH* Mutations Implicated in CVD

Among the naturally occurring mutations of the human *XDH* gene identified, the majority are characterised by a reduction or absence of enzymatic activity (Table 1). A smaller subset, however, have been associated with increased enzymatic activity or have been positively associated with CVD risk factors such as hypertension (Table 2). Interestingly, there is evidence to suggest that the prevalence of these mutations depends upon ethnicity and below we summarise the main findings for key groups.

### 5.1. XDH Mutations and Hypertension in Japanese Populations

There is evidence linking *XDH* mutations with the development and progression of CVD risk factors in Japanese cohorts, including hypertension, atherosclerosis, and chronic kidney disease (CKD) [91]. Sequencing of the *XDH* gene (including the promoter region and 36 exons) in 48 randomly selected hypertensive Japanese patients (from a 953-patient cohort) identified three heterozygous missense mutations; 9 patients: Gly172Arg, 1 patient: Ala932Thr, and 3 patients: Asn1109Thr—along with eight common SNPs mutations. Subsequent genotype screening of a larger cohort of 953 Japanese hypertensive patients and 1818 Japanese individuals in the Suita Study, identified one homozygous Gly172Arg carrier and three homozygous Asn1109Thr carriers. These four carriers, from the hypertensive cohort, all had resistant hypertension despite being on antihypertensive drug intervention. However, only one patient with Asn1109Thr exhibited hyperuricemia and hyperlipidaemia was observed in one Gly172Arg and two Asn1109Thr carriers [91].

Initial association studies within a cohort of 1818 Japanese individuals highlighted several *XDH* SNPs linked to hypertension, showing distinctions between males and females. In men, the exonic SNP 47686C>T (resulting in a synonymous substitution, Ile737Ile) and the intronic SNP 69901A>C, both in recessive form, were significantly associated with hypertension, with odds ratios of 1.52 (95% CI, 1.01–2.29) and 3.14 (95% CI, 1.06–9.27), respectively. Additionally, the dominant exonic SNP 67873A>C (Asn1109Thr) showed an odds ratio of 1.84 (95% CI, 1.11–3.06). Female participants exhibited associations only with the SNP 69901A>C, where carriers of AC + CC alleles had a systolic blood pressure 2.75 mmHg higher than AA carriers. The Asn1109Thr mutation (SNP 67873A>C) also showed associations with elevated diastolic BP by 2.75 mmHg in AC + CC carriers compared to WT *XDH* alleles. These findings suggest that genetic variations in *XDH* may contribute to BP regulation in a sex-dependent manner, certainly within this Japanese cohort.

Further analyses assessed associations between *XDH* SNPs and carotid atherosclerosis. Only the intronic SNP 69901A>C, which had previously shown associations with elevated BP in both sexes, was also linked to carotid atherosclerosis. This association remained significant even after adjusting for key cardiovascular risk factors, such as systolic and diastolic BP, BMI, smoking status, diabetes, and dyslipidaemia. Despite highlighting Ala932Thr and Asn1109Thr as potential risk factors for hypertension, this study lacked functional analyses to elucidate the mechanisms by which these genetic variants might impact BP or vascular health. Furthermore, whether these associations are relevant to different populations should be considered, particularly since both ethnicity and/or the environment impact XOR expression and activity [92,93].

### 5.2. XDH SNPs and Hypertension in Chinese Populations

In a rural Chinese cohort, a case-control study by Wu et al. genotyped five selected tag SNPs and identified three SNPs significantly associated with hypertension: rs1042039, rs1054889, and rs2073316 [94]. Notably, rs1042039 and rs1054889 are located in the 3′-untranslated region (3′-UTR), which influences mRNA stability and translation efficiency, potentially impacting protein expression levels. These findings suggest that non-coding SNPs may also modulate *XDH* gene expression and influence BP (Table 3). However, in the absence of larger clinical studies or functional characterisation, caution should be taken in interpreting these regulatory SNPs as definitive risk factors for CVD.

### 5.3. XDH Mutations and Hypertension in European Populations

In a European case-control cohort [95] comprised of 185 hypertensive patients and 385 controls, two *XDH* SNPs (-337GA, rs206812; and 565 + 64CT, rs2073316) were examined for their association with hypertension. The hypertensive patients exhibited higher BMI, fasting glucose, total cholesterol, triglycerides, and high sUA compared to controls. The two SNPs were in linkage disequilibrium, and analyses found that 565 + 64CT was positively associated with both systolic and diastolic BP in hypertensive patients, whereas 337GA was associated only with systolic pressure. Notably, both SNPs exhibited additive effects, increasing systolic pressure by 35 mmHg and diastolic pressure by 12 mmHg in homozygous mutants compared to WT *XDH* alleles.

Despite the mutants displaying a positive association with hypertension, no corresponding association with UA levels was identified. This suggests that these mutations may influence processes other than the enzymatic oxidative capacity, which would typically result in elevated UA production. In an independent sample of 100 hypertensive patients, the same SNPs were investigated for correlations with oxidative stress markers, malondialdehyde (MDA) and 8-oxo-deoxoguanosine (8-oxo-dG). Homozygous carriers of both SNPs exhibited a significantly positive correlation with both oxidative stress markers, implicating increased ROS production. Taken together, these results suggest that the SNPs may increase XOR activity, leading to excess ROS, which then causes lipid and DNA oxidation, as evidenced by increased levels of MDA and 8-oxo-dG, ultimately inducing cellular dysfunction and death. However, given that these mutations are located outside the coding region, further biochemical studies are needed to confirm and clarify their mechanistic impact on BP regulation.

**Figure 4:**
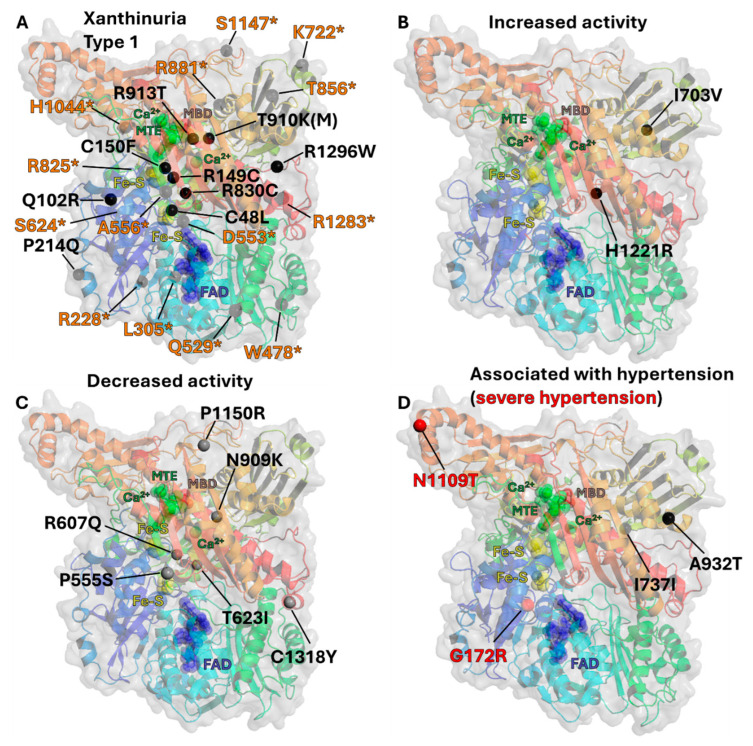
Position of residue mutations which induce a phenotype. (**A**) Residues indicate sites of non-synonymous and non-sense mutations associated with Xanthinuria Type 1. * highlights non-sense mutation site residues (Orange) as presented in Table 1 (**B**) Residues indicate sites of non-synonymous mutations associated with increased XOR activity. (**C**) Residues (Gray) indicate sites of non-synonymous mutations associated with decreased XOR activity. (**D**) Residues indicate sites of non-synonymous and synonymous mutations that are associated with (Black) hypertension, or (Red) severe hypertension. Figure derived from human XOR crystal structure (PDB 2E1Q). Also shown are (Green) substrate MTE in the molybdenum active pocket, (Yellow) Fe-S clusters, (Blue) FAD in the FAD domain, and (Brown) molybdenum (MBD).

In a longitudinal study by Scheepers et al., a random cohort of 2769 Europeans [96], including 719 patients hypertensive at baseline, was examined to assess associations between 28 *XDH* SNPs and hypertension risk over an 8–9 year follow-up period. The selected SNPs had minor allele frequencies (MAF) exceeding 1%, ranging from 1.7% to 48.6%. Of the 28 SNPs, 25 complied with Hardy–Weinberg equilibrium and were included in the genotyping analyses, although these SNPs were not in linkage disequilibrium with each other. The association of these SNPs with the risk of developing hypertension over 8–9 years identified that three SNPs, rs1190443, rs148756340, and rs2043013, were significantly associated with high BP. Similarly, carriers of the non-minor allele for rs148756340 had a 70% higher risk of becoming hypertensive. For the third SNP, rs2043013, minor allele carriers demonstrated a mean arterial pressure increase of 4.8 mmHg. While these findings reinforce associations between *XDH* SNPs and BP, no accompanying biochemical analyses were performed to clarify the mechanisms underlying these intronic SNP impact. Further biochemical investigation is warranted to better understand these associations and potential pathways influencing BP (see Figure 4).

**Table 3:** Location of non-coding SNPs associated with hypertensive phenotypes *. The nucleotide reference sequence used to indicate codon changes are derived from the individual primary research paper indicated.

Domain	Codon Change *	Hypertension (HP) Phenotype	Reference
Promoter	c.-337G>A	Association with HP	[95]
Intron	c.565 + 64T>C	Association with HP	[95]
Intron	c.69901 A>C	Association with HP in men	[91]
Intron	c.27712 T>C	Association with HP	[96]
Intron	c.35337 T>C	Association with HP	[96]
Intron	c.31607275A>G	Association with HP	[96]
Intron	c.31369457A>C	Association with HP	[96]
Intron	c.31388163 G>A, G>C	Association with HP	[94]
3′-UTR	c.31335440T>C	Association with HP	[94]
3′-UTR	c.31334442 G>A, G>C, G>T	Association with HP	[94]

## 6. Biochemical Impact of Human *XDH* Mutations

### 6.1. Impact of XDH Mutation on XOR Expression

The first study investigating the impact of non-synonymous genetic mutations of human *XDH* on XOR enzymatic activity was published by Kudo et al. in 2008 [85]. In this study, the authors sequenced the entire coding region of *XDH* from 96 unrelated healthy Japanese individuals, identifying both known mutations and a novel non-synonymous mutation, His1221Arg (Figure 4). To study the effect of these mutations upon enzymatic activity, mutant and WT proteins were transiently expressed in COS-7 cells, with protein expression assessed by immunoblotting. Notably, mutants Asn909Lys, Thr910Lys, Asn1109Thr, His1221Arg, and Pro555Ser exhibited markedly lower XOR protein expression levels, compared to WT *XDH*. Whilst these differences were not quantified, it suggests that these mutations may influence protein maturation or stability and/or mRNA stability.

In our recent study, we observed similar differences in expression and quantified them at both protein and mRNA levels. Our findings corroborated much of the work of Kudo et al.; however, we also identified additional functional insights. For instance, Kudo et al. reported that the Asn909Lys mutation produced almost undetectable levels of protein (expressed in COS-7 cells), whilst, in contrast, we showed that expression in HEK2937T cells, although lower than WT *XDH*, was still significantly higher than the untransfected control [16]. This discrepancy may relate to the different experimental approaches or cell-specific expression effects. This hypothesis is supported by Kudos findings using a luciferase reporter assay to evaluate the impact of *XDH* promoter SNP mutations on XOR expression across different cell lines [97].

### 6.2. Impact of XDH Mutations on Enzymatic Activity

Kudo et al. measured UA production in *XDH* mutant-expressing cell homogenates exposed to increasing concentrations of xanthine (5–30 µmol/L) [85]. In this work, they demonstrated that mutations His1221Arg and Ile703Val significantly increase Vmax or Vmax/km compared to WT, affecting the oxidation rate of hypoxanthine to xanthine and xanthine to UA. Despite these mutations occurring at the Mo-Pt domain surface, not within the active site cavity (Figure 4), Nishino et al. speculated that they accelerate UA release from the Mo-Pt domain (considered the rate-limiting step), thus enhancing enzyme efficiency [98].

In our studies we demonstrated that His1221Arg, but not Ile703Val, significantly increases the production of O_2_^•−^ compared to WT in the presence of xanthine but not NADH (a reducing agent acting at the FAD site) [16]. We also showed that these non-synonymous mutations, in particular His1221Arg, induced post-translational modifications that shifted the proportions of the enzyme in the XDH isoform (NADH-producing) to the XO isoform (ROS-producing). This may explain the increased UA and O_2_^•−^ production by cells expressing the His1221Arg mutant. More importantly, in the presence of NO_2_^−^, His1221Arg demonstrated increased NO_2_^−^ reductase capacity and subsequently increased ·NO generation compared to WT. We speculated that dietary NO_3_^−^ could be used to increase the levels of NO_2_^−^ available as a substrate converting XOR from a pro-oxidative to a NO-generating phenotype. We previously referred to this process as “repurposing of XOR” [99], and this repurposing may be enhanced in carriers of pro-oxidative mutations like His1221Arg.

The two catalytic domains of XOR, Mo-Pt and FAD, can be considered pharmacologically independent. DPI inhibits all reactions at the FAD site, whereas febuxostat and allopurinol block purine oxidation and NO_2_^−^ reduction occurring at the Mo-Pt domain [24,27]. Genetic mutations affecting the Mo-Pt domain may similarly result in impaired purine oxidation while leaving the FAD domain functional. For instance, we recently demonstrated [16] that a mutation occurring at the Mo-Pt domain, Asn909Lys, reduces XOR oxidising capacity towards xanthine but retains NADH oxidation at the FAD site, enabling O_2_ reduction to O_2_^−^ and H_2_O_2_. In the absence of any reducing agent, this mutation showed comparable NO_2_^−^-reductase activity to human WT XOR, suggesting that while the mutation impairs purine interaction at the Mo-Pt domain, it does not affect interaction with NO_2_^−^. Similarly, a double mutation of Trp336 and Phe337, located within the enzyme’s highly packed relay system, resulted in reduced oxidative capacity with xanthine as the reducing agent but increased activity when NADH was used [16]. However, mutated Trp336/Phe377 XOR demonstrated a significantly greater NO_2_^−^ reductase activity compared to WT XOR.

To date, only two studies—Kudo et al. in 2008 and our more recent study—have functionally (biochemically) investigated naturally occurring XOR mutations. Findings from these studies, particularly the latter, suggest that a comprehensive understanding of non-synonymous mutations in XOR requires assessing in terms of not only its well-documented activities in UA and ROS production but also its lesser-known, potentially more biologically important, NO_2_^−^-reductase activity. For example, the His1221Arg mutation, which significantly increases UA production and enhances both O_2_^·^^−^ and NO_2_^−^ reduction activities, represents a mutation where precision medicine approaches may hold promise. In this context, NO_3_^−^ supplementation could exploit the increased NO_2_^−^-reductase capacity associated with this mutation, converting the pro-oxidative state into a NO-generating phenotype, potentially mitigating cardiovascular risk and also benefitting patients with established CVD. Conversely, mutations like Asn909Lys and the double mutation of Trp336/Phe337, while retaining NO_2_^−^ reductase activity, show selective deficits in xanthine oxidation. For such mutations, NO_2_^−^ supplementation may also be beneficial as they maintain some ability to generate NO from NO_2_^−^ even when purine oxidation is compromised. These examples underscore the importance of evaluating the specific effects of each mutation on the multifaceted catalytic activities of the enzyme, guiding the development of individualised therapeutic strategies. Conversely, patients with mutations exhibiting limited NO_2_^−^ reductase activity are unlikely to benefit from NO_3_^−^/NO_2_^−^ supplementation. For these cases, treatment with an XOR inhibitor, like febuxostat or allopurinol, might be more suitable, as these mutations display reduced capacity to produce NO and may not be linked to an elevated risk of mortality associated with XOR inhibition [100]. This tailored approach acknowledges the nuanced impact of specific genetic mutations, paving the way for more effective and individualised therapeutic interventions in managing cardiovascular disease. Whilst the above supports the hypothesis that XOR could be the subject of a precision medicine approach, we believe that further pre-clinical studies (both further biochemical assessment and in vivo models) are required.

**Figure 5:**
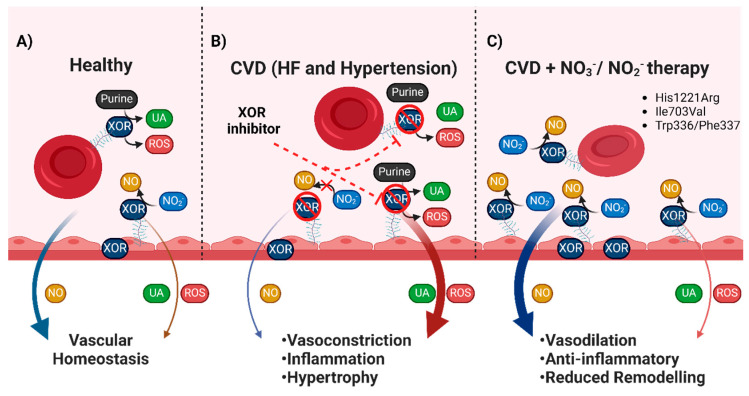
Vascular localisation of XOR in health and disease. (**A**) Healthy individuals have modest XOR expression in the vasculature which is either extracellularly bound to glycosaminoglycans (GAGs) in the vessel lumen, or intracellularly expressed in endothelial cells. Healthy individuals have a relative balance between XOR NO_2_^−^ reductase capacity and production of UA and ROS which contribute to vascular homeostasis. (**B**) In CVD, upregulated expression and activity of RBC and endothelial XOR results in enhanced UA and ROS production. The increased production of UA and ROS contribute to attenuate NO signalling and promotes vasoconstriction, inflammation, and cardiac hypertrophy. CVD patients with XOR mutations that impair NO_2_^−^ reductase activity may not experience increased CVD risk with febuxostat administration. In conjunction, they will not be able to utilise NO_3_^−^/NO_2_^−^ intervention and may not have any benefit from the repurposing of the enzyme. (**C**) The elevated XOR expression and activity in CVD patients could alternatively be exploited, via NO_3_^−^ and NO_2_^−^ therapy. Increased levels of exogenously derived NO_2_^−^ could restore the attenuated NO signalling and reduce UA and ROS production. This intervention could be more beneficial in patients that harbour mutants (e.g., His1221Arg, and Ile703Val) with enhanced NO_2_^−^ reductase capacity. This could convert mutants that may have greater propensity to generate ROS and UA from pathogenic to disease resolving. Overall, this could then help stratify CVD patients for which XOR intervention may be appropriate—inhibition or exploitation ensuring reduced risk with XOR inhibitors or efficacy with dietary NO_3_^−^ intervention.

In addition to mutations specifically influencing XOR activity these mutations also have the potential to modify protein structure and spatial re-arrangement, potentially impacting its capacity to interact with glycosaminoglycans (GAGs). This possibility is a major issue for the CV system, since the vast majority of XOR within this system is derived from enzyme released from the liver into the circulation with its consequent binding at sites (predominantly the endothelium and erythrocytes) distinct from its site of synthesis. Below, we discuss in more detail the potential importance of this issue (Figure 5).

## 7. Future Horizons: Unravelling the Impact of XOR Genetic Mutations upon GAG/Proteoglycan Interactions

While some basic enzymatic properties of naturally occurring XOR mutations have been characterised in vitro, this approach limits our ability to translate these findings to the complex in vivo environment. In CVD, GAGs sequester upregulated systemic XOR expression, particularly in endothelial and red blood cells [11,101]. Thus, the in vivo milieu offers the potential to amplify/alter the functional changes of XOR genetic mutants due to the known interaction of the enzyme with GAGs. Therefore, the potential influence of these genetic mutations in XOR (Figure 5) on its interactions with secondary structures, notably GAGs and proteoglycan superstructures [102], requires assessment.

**Figure 6:**
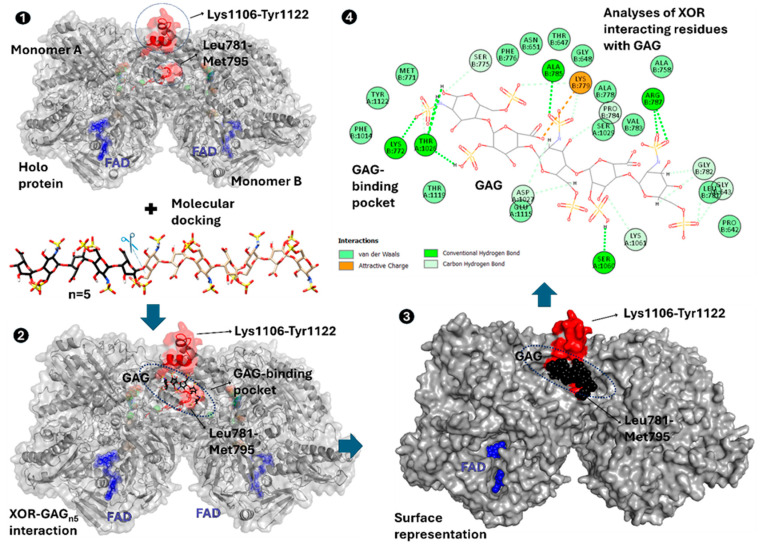
Preliminary in silico docking experiments of XOR–GAG interactions. Workflow of experimental approach: (**1**) Highlighting areas of XOR residues (Leu781-Met795 and Lys1106-Tyr1122) and areas that have been associated with GAG interactions for docking of a small heparin structure (PDB:1HPN was truncated to n = 5 residues); (**2**) The results of preliminary docking experiments of the heparin (black) fragment into regions of interest (red); (**3**) Demonstrates the surface representation of the heparin fragment docking into a well-defined interface pocket; (**4**) Residues Lys772, Ser775, Lys779, Leu781, Gly782, Pro784, Ala785, Arg787, Thr1026, Asp1027, Ser1060, Lys1061, Glu1115, Thr1119, and Tyr1122 form strong electrostatic interactions with the sulfonate groups on heparin. Therefore, any genetic mutations that have the capacity to interfere with these key residues could impact the binding, stability, and affinity of GAGs to XOR. Colored Blue is FAD in the FAD domain.

The Mo-Pt domain of XOR contains highly conserved lysine and arginine residues at positions Leu781-Met795 and Lys1106-Tyr1122 [103,104]. These positively charged domains interact with negatively charged heparin and heparan sulphate, with higher affinity for heparin. The conserved Lys1106-Tyr1122 domain is present in many signalling molecules that interact with GAGs. Consequently, circulating plasma XOR can bind to vascular endothelial cells via high-affinity electrostatic interaction with GAGs on the luminal glycocalyx (K_d_ = 6 nM) [105] (see Figure 6).

Immobilisation by GAGs alters XOR kinetics due to changes in substrate affinity. Thus, mutations inducing spatial rearrangement of the Mo-Pt domain could alter XOR affinity for GAGs and subsequently change the kinetics of the mutated protein. For instance, the purine substrate xanthine has a ~3-fold decreased affinity for GAG-bound bovine XOR [106]. In this study the authors also showed that GAG-bound bovine XOR expresses a ~5-fold increase in inhibition constant (K_i_) for allopurinol/oxypurinol (bound: 451 nM vs. unbound: 85 nM). Interestingly, whilst the efficacy of allopurinol/oxypurinol was reduced by GAG-binding, the efficacy of febuxostat has been shown to be largely retained. Indeed, GAG-bound XOR was shown to cause a small ~2.5-fold increase in febuxostat IC_50_ for UA generation and ~5-fold for O_2_^•−^ generation, whereas with allopurinol the IC_50_ was shown to increase 22-fold when GAG-bound [107].

The limited impact of GAG-binding upon the inhibitory activity of febuxostat has been attributed to the fact that febuxostat binding to XOR does not depend upon covalent bond formation with the reduced state molybdenum (Mo^+IV^), as is the case for allopurinol/oxypurinol [108,109]. Instead, febuxostat utilises conserved XOR phenylalanine residues (Phe914, Phe1009, Phe1013) and leucine residues (Leu873, Leu1014) for multiple weak hydrophobic interactions [110]. Both febuxostat and allopurinol interact with Arg880 and Glu802 via hydrogen bonds, but only allopurinol forms an additional hydrogen bond with Glu1262 [17]. Febuxostat rotates between its ring structures allowing greater flexibility in integrating into the XOR active site. This suggests that structural differences in the efficacy of XOR antagonists when XOR is GAG-bound offer potential insights into how genetic mutations could alter the 3D structure and function of XOR and are worthy of further investigation.

In addition to differences in the Mo-site characteristics of the site to which the XOR inhibitors bind GAG-bound, XOR also shows altered FAD kinetics causing increased two-electron reduction of O_2_ (producing H_2_O_2_ > O_2_^•−^) [107]. If a genetic mutation with elevated O_2_^•−^ generation (His1221Arg) impairs GAG binding, it could enhance the ability of the mutated XOR to generate ROS [16]. To date, there has been little assessment of the influence of GAG-bound XOR upon its NO_2_^−^ reductase capacity, with a single study demonstrating that GAG-bound XOR increased NO_2_^−^ reductase capacity versus free XOR [27]. This increased capacity was linked to a decreased purine affinity and a ~3-fold increase in affinity for NADH at the FAD domain [111]. Acidic pH, which favours XOR-dependent NO_2_^−^ reductase activity, also leads to increased affinity for heparin-Sepharose [112]. However, basic pH favours XOR binding to cells, indicating a need to discriminate how full proteoglycan structures influence XOR binding [105]. Genetic mutations that interfere with GAG–XOR binding could increase the proportion of free plasma XOR, favouring ROS generation over NO production.

Preliminary studies on XOR and GAG interactions often use heparan sulphate sepharose beads in column chromatography, which do not replicate in vivo environments with varying substrate availability. In vitro assays typically use exogenous bovine XOR, which, when applied excessively to vascular endothelial cells, increases ROS generation and attenuates NO/cGMP signalling in smooth muscle cells [105]. However, using these systems it is very difficult to determine the exact localisation of XOR and whether the ROS is generated via bound XOR/endocytosed XOR or excess unbound exogenous XOR. Thus, technological advances enabling such discrimination are needed before a firm establishment of the relative contributions of the bound versus unbound forms of XOR can be clarified.

As we have shown, mutations in XOR alter expression levels [16]. Structural changes in the protein due to these mutations that alter proteoglycan binding could alter plasma XOR expression by modulating internalisation, recycling, degradation, and endocytosis. Studies suggest that incubation of XOR with endothelial cells after some time will lead to some internalisation, but the role of proteoglycans in this pathway remains unclear. Chondroitin sulphate proteoglycans have been shown to play a greater role than heparan sulphate proteoglycans in XOR internalisation into either bovine or human endothelial cells [105]. Internalisation of proteoglycans is typically clathrin-mediated via proteoglycan internal PDZ domain interaction with Arf6/phosphoinositide (PIP2) [113]. Genetic variants could change intracellular XOR compartmentalisation, recycling, and inactivation. Mutations decreasing endocytosis could lead to extracellular XO accumulation. Stress-induced hepatic XOR exocytosis depends on lysosomal trafficking but the association with proteoglycans is unclear [114]. Lysosomal compartmentalisation could modulate XOR activity due to the environmental acidic pH that favours NO_2_^−^ reductase activity and localised substrate availability (decreased ROS generation) [115]. Increased proteoglycan endocytosis may explain why type 2 diabetic patients with CVD have decreased plasma XOR expression versus patients without CVD [116].

In addition to understanding the impact of genetic mutations on GAG/proteoglycan-mediated binding and XOR activity, the impact of impaired/increased XOR binding on GAG/proteoglycan activity is less established. The endothelial glycocalyx regulates many vascular functions, such as permeability, inhibition of platelet and immune cell activity, and signal transduction [117]. Genetic mutations increasing O_2_^•−^ potential (His1221Arg) can increase endothelial glycocalyx shedding. Increased proteoglycan shedding occurs in acute and chronic inflammation settings, where elevated purine availability drives ROS generation in GAG-bound XOR.

The assessment of the potential impact of these mutations in CVD reveals a potential intricate interplay between XOR, substrate concentration, GAGs, and proteoglycans. These interactions affect the kinetics, antagonist responses, and intracellular processes of XOR with implications for ROS generation and NO production in CV settings. While most research assessing XOR enzymatic activity has been conducted mainly in controlled in vitro settings with isolated XOR, future studies should explore complex physiological conditions to bridge the gap between laboratory findings and clinical relevance. Additionally, the influence of genetic mutations upon XOR internalisation, recycling, and proteoglycan binding remains an area for further investigation.

## 8. Conclusions

In summary, recent functional assessment of the impact of XOR genetic mutations on function has revealed new biology that emphasises the importance of assessing all of the three biochemical functions of the enzyme to fully understand the significance of the mutation. We propose that further functional characterisation of *XDH* mutants may unveil why sUA has not universally translated as a marker of CVD. Furthermore, we postulate that understanding the functional NO_2_^−^ reductase activity of *XDH* mutants, in tandem with pharmacogenomic testing, could assist clinicians in determining whether XOR inhibition or XOR repurposing via NO_3_^−^/NO_2_^−^ supplementation is appropriate.

The complex relationship between XOR genetic mutations, GAGs, and proteoglycans presents an exciting avenue for future research in CVD. A comprehensive understanding of these interactions could lead to improved insights into the mechanisms underlying disease and potential therapeutic interventions targeting XOR and its genetic variants. This ongoing exploration holds the promise of enhancing our knowledge of cardiovascular health and disease, ultimately benefiting patient outcomes.

## Figures and Tables

**Figure 3:**
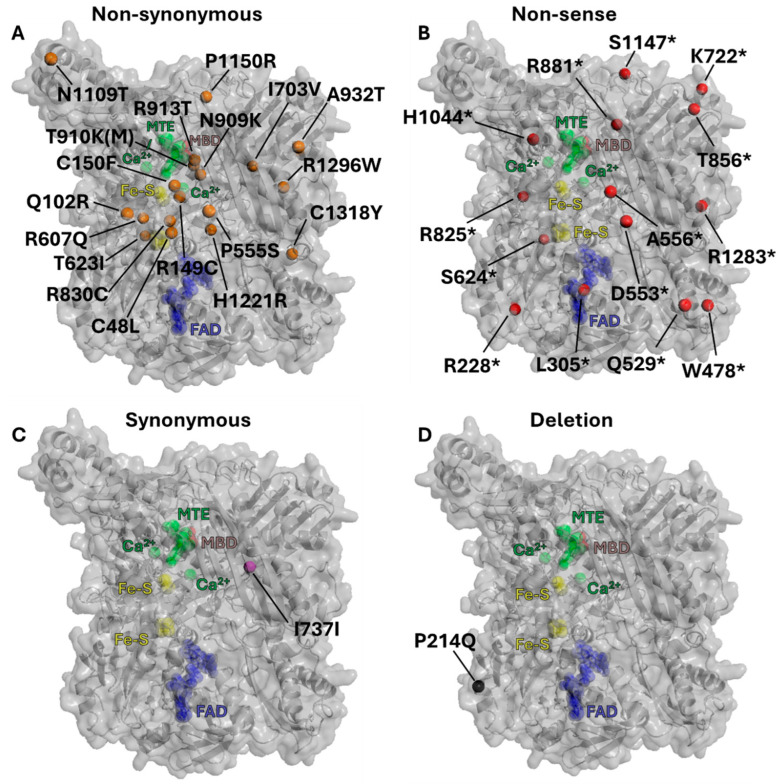
Position of residue mutations which attenuate XOR activity. (**A**) Orange residues indicate sites of non-synonymous mutations. (**B**) Red residues indicated by * represent sites of non-sense mutations that also mediate Xanthinuria Type 1 as presented in Table 1. (**C**) Purple residue indicates a site of synonymous mutation. (**D**) Black residue indicates a site of deletion mutation. Figure derived from human XOR crystal structure (PDB 2E1Q). Also shown are (Green) substrate MTE in the molybdenum active pocket, (Yellow) Fe-S clusters, (Blue) FAD in the FAD domain, and (Brown) molybdenum (MBD).

**Table 1:** Location of SNPs associated with attenuated XOR activity and Xanthinuria Type I. * The nucleotide reference sequence used to indicate codon changes is derived from the individual primary research paper indicated. Orange indicates non-synonymous mutations as shown in Figure 3A. Red indicates non-sense mutations as shown in Figure 3B.

Domain	Codon Change *	Codon Number	Amino Acid Change	Type Mutation	Phenotype	References
Fe/S cluster	c.140_141insG	47	Cys48Leufs*12	Non-synonymous	Xanthinuria Type I	[76]
Fe/S cluster	c.305A>G	102	Gln102Arg	Non-synonymous	Xanthinuria Type I	[77]
Fe/S cluster	c.445C>T	149	Arg149Cys	Non-synonymous	Xanthinuria Type I	[78]
Fe/S cluster	c.449G>T	150	Cys150Phe	Non-synonymous	Xanthinuria Type I	[79]
Linker	c.641del_C	214	Pro214Glnfs*4	Deletion	Xanthinuria Type I	[80,81]
FAD	c.682C>T	228	Arg228 *	Non-sense	Xanthinuria Type I	[82]
FAD	c.913del_	304	Leu305*1	Non-sense	Xanthinuria Type I	[79]
FAD	c.1434G>A	478	Trp478 *	Non-sense	Xanthinuria Type I	[79]
Mo-Pt	c.1585C>T	529	Gln529 *	Non-sense	Xanthinuria Type I	[75]
Mo-Pt	c.1658_1659Ins_C	553	Asp553Ala fs*17	Non-sense	Xanthinuria Type I	
Linker	c.1663C>T	555	Pro555Ser	Non-synonymous	Decreased Activity	[83]
Linker	c.1664_1665Ins_C	556	Ala556Ser *fs15	Non-sense	Xanthinuria Type I	[84]
Mo-Pt	c.1820G>A	607	Arg607Gln	Non-synonymous	Decreased Activity	[83]
Mo-Pt	c.1868C>T	623	Thr623Ile	Non-synonymous	Decreased Activity	[83]
Mo-Pt	c.1871C>G	624	Ser624 *	Non-sense	Xanthinuria Type I	[79]
Mo-Pt	c.2164A>T	722	Lys722 *	Non-sense	Xanthinuria Type I	[85]
Mo-Pt	c.2473C>T	825	Arg825 *	Non-sense	Xanthinuria Type I	[81]
Mo-Pt	c.2488C>T	830	Arg830Cys	Non-synonymous	Xanthinuria Type I	[86]
Mo-Pt	c.2567del_C	856	Thr856Lys fs*73	Non-sense	Xanthinuria Type I	[82,87]
Mo-Pt	c.2641C>T	881	Ar881*	Non-sense	Xanthinuria Type I	[81]
Mo-Pt	c.2727C>A	909	Asn909Lys	Non-synonymous	Decreased Activity	[83]
Mo-Pt	c.2729C>A	910	Thr910Lys	Non-synonymous	Xanthinuria Type I	[83]
Mo-Pt	c.2729C>T	910	Thr910Met	Non-synonymous	Xanthinuria Type I	[76,88]
Mo-Pt	c.2737C>T	913	Arg913Thr	Non-synonymous	Xanthinuria Type I	[89]
Mo-Pt		1044	His1044 fs*12	Non-sense	Xanthinuria Type I	
Mo-Pt	c.3440C>G	1147	Ser1147 *	Non-sense	Xanthinuria Type I	
Mo-Pt	c.3449C>G	1150	Pro1150Arg	Non-synonymous	Decreased Activity	[83]
Mo-Pt	c.3847C>T	1283	Arg1283 *	Non-sense	Xanthinuria Type I	[90]
Mo-Pt	c.3886C>T	1296	Arg1296Trp	Non-synonymous	Xanthinuria Type I	[78]
Mo-Pt	c.3953G>A	1318	Cys1318Tyr	Non-synonymous	Decreased Activity	[83]

**Table 2:** Location of coding SNPs associated with hypertensive phenotypes * The nucleotide reference sequence used to indicate codon changes is derived from the individual primary research paper indicated. Orange indicates non-synonymous mutations as shown in Figure 3A. Purple indicates synonymous mutations as shown in Figure 3C.

	Codon Change *	Codon Number	Amino Acid Change	Type Mutation	Hypertension (HP) Phenotype	Reference
Fe/S-FAD Linker	c.514 G>A	172	Gly172Arg	Non-synonymous	Severe HP	[91]
Mo-Pt	c.2107 A>G	703	Ile703Val	Non-synonymous	Increased activity	[85]
Mo-Pt	c.47686 C>T	737	Ile737Ile	Synonymous	HP in men	[91]
Mo-Pt	c.2794 G>A	932	Ala932Thr	Non-synonymous	HP in men	[91]
Mo-Pt	c.3326 A>C	1109	Asn1109Thr	Non-synonymous	Severe HP	[91]
Mo-Pt	c.3662 A>G	1221	His1221Arg	Non-synonymous	Increased activity	[85]
